# Enlargement of the human prefrontal cortex and brain mentalizing network: anatomically homogenous cross-species brain transformation

**DOI:** 10.1007/s00429-025-02896-7

**Published:** 2025-01-24

**Authors:** Hideki Amano, Hiroki C. Tanabe, Naomichi Ogihara

**Affiliations:** 1 Department of Biological Sciences, Graduate School of Science, The University of Tokyo, Tokyo, 113-0033 Japan; 2 Department of Cognitive and Psychological Sciences, Graduate School of Informatics, Nagoya University, Nagoya, 464-8601 Japan

**Keywords:** Brain evolution, Human and chimpanzee brains, Computational neuroanatomy, Segmentation, Voxel-based morphometry

## Abstract

To achieve a better understanding of the evolution of the large brain in humans, a comparative analysis of species differences in the brains of extant primate species is crucial, as it allows direct comparisons of the brains. We developed a method to achieve anatomically precise region-to-region homologous brain transformations across species using computational neuroanatomy. Utilizing three-dimensional neuroimaging data from humans (*Homo sapiens*), chimpanzees (*Pan troglodytes*), and Japanese macaques (*Macaca fuscata*), along with the anatomical labels of their respective brains, we aimed to create a cross-species average template brain that preserves neuroanatomical correspondence across species. Homologous transformation of the brain from one species to another can be computed using the cross-species average brain. Applying this transformation to human and chimpanzee brains revealed that, compared to chimpanzees, humans had significantly larger and more expanded prefrontal cortex, middle and posterior temporal gyrus, angular gyrus, precuneus, and cortical areas associated with mentalization. This neuroanatomically homologous brain transformation enables the systematic investigation of the similarities and differences in brain anatomy and structure across different species.

**Supplementary Information:**

The online version contains supplementary material available at 10.1007/s00429-025-02896-7.

## Introduction

The evolution of the disproportionally large brain in modern humans remains a key question in paleoanthropology. Studies on the evolutionary changes in the endocranial volume (as a proxy of the brain size) indicated that the evolutionary expansion of the brain started approximately 2–2.5 million years ago, when the genus *Homo* emerged from an early hominin (*Australopithecus*), whose brain size remained similar to that of modern chimpanzees (Holloway 2008). However, the brain itself has not been fossilized, and details of the selective pressures modulating the evolution of large brains in the human lineage remain unclear, mainly because of the inability to estimate the evolutionary changes in cognitive and social abilities from the endocasts of fossil hominins.

To achieve a better understanding of the evolution of the large brain in humans, a comparative analysis of species differences in the brains of extant primate species is crucial, as it allows direct comparisons of the brains using neuroimaging, cytoarchitectural, and histological techniques (e.g., Van Essen et al. 2019; Reyes et al. 2023; Amiez et al. 2021). Comparisons of the human brain with those of the chimpanzee and macaque are of particular importance because the chimpanzee is the closest extant relative of humans, and the macaque is a non-hominoid anthropoid whose brain structure is less specialized (derived) with respect to the human and chimpanzee brains (Preuss 2000), but it is the primate species with the most well-studied brain neuroanatomy and cognitive mechanisms as a model for the human brain (Passingham 2009).

In the field of neuroimaging, efforts have been made to computationally quantify the structural changes in each brain region using magnetic resonance imaging (MRI) in the voxel-based morphometry (VBM; Wright et al. 1995; Ashburner and Friston 2000) method. VBM enables automatic measurement and comparison of each brain region on a voxel-by-voxel basis based on the spatial normalization process, which involves volumetric registration (warping) of MRI scans of the heads from different individuals to the population-averaged target or template brain based on a series of linear and nonlinear spatial transformations (DARTEL algorithm; Ashburner 2007). If the template brain is parcellated into brain regions and labeled using digital atlases of the human brain (e.g., Tzourio-Mazoyer et al. 2002), the change in the volume of each brain region can be calculated using the transformation function. Although the segmentation of these atlases was achieved based on macroscopic gyral and sulcal morphology, it has been suggested that the borders of cytoarchitectural areas often correspond to sulcal morphology (White et al. 1997). This indicates that such brain atlases can be utilized to parcellate brain regions with reasonable accuracy and precision. Therefore, the VBM technique has been widely used to investigate and successfully evaluate brain changes in neurological disorders, such as neurodegenerative diseases (Whitwell and Jack 2005) in humans.

Although VBM can be applied to identify detailed differences in brain structure and cortical organization across the three species, this is unfeasible because the brain shapes of the three species vary widely. For example, in the human brain, the inferior frontal gyrus is adjacent to the lateral fissure and insula; however, in the macaque brain, these structures are nonadjacent (Zilles and Amunts 2018). Such large differences in the topographic organization of brain regions make the calculation of the anatomically homologous average brain of the three distinct species rather challenging with conventional methodology that solely relies on the DARTEL algorithm.

Cross-species differences in cortical organization have been investigated using surface-based registration between chimpanzees and humans (Hopkins et al. 2017; Wei et al. 2019), macaques and humans (Van Essen and Dierker 2007; Xu et al. 2020), as well as across all three species (Eichert et al. 2020). Hopkins et al. (2017) and Wei et al. (2019) used the human anatomical atlas (Desikan–Killiany atlas; Desikan et al. 2006) to parcellate the chimpanzee brain because no chimpanzee brain atlas was available at that time; however, each cortical region may not have been homologously segmented for the chimpanzee brain. Eichert et al. (2020) achieved area-to-area homologous registration using species-average myelin maps, that is, the ratio of T1- and T2-weighted structural images presumed to represent the functional specialization of brain regions (Van Essen et al. 2018). Therefore, the cortical and brain regions were not parcellated, and the gross proportion of cortical and brain regions could not be compared across species. Van Essen and Dierker (2007) and Xu et al. (2020) used homologous landmarks defined on the brain surface for topologically equivalent surface-based registration. However, chimpanzees were not included in the comparison. Cross-species differences in cortical organization have also been explored using diffusion MRI tractography, which identifies topologically equivalent gray matter regions based on the connectivity patterns of white matter tracts (Mars et al. 2018; Eichert et al. 2019). However, these studies did not include chimpanzees in their analyses.

In the present study, we aimed to develop a computational neuroanatomy-based method to achieve an anatomically rigorous, region-to-region homologous transformation of the brain from one species to another among the three species. Specifically, using the 3D neuroimaging data of (1) humans (*Homo sapiens*), (2) chimpanzees (*Pan troglodytes*, African great ape), and (3) Japanese macaques (*Macaca fuscata*, non-hominoid anthropoid), and the anatomical labels of their respective brains, we attempted to create a cross-species average template brain with preserved interspecies neuroanatomical correspondence of brain regions. If this can be achieved, the homologous transformation of the brain of one species to another can be computed using the cross-species average brain. Such an interspecies neuroanatomically homologous brain transformation enables the systematic investigation of similarities and differences in brain anatomy, structure, or function across different species, and potentially serves as a fundamental technology for estimating the regional morphology of the brain in fossil hominins.

## Materials and methods

### Samples

We obtained T1-weighted whole-brain magnetic resonance structural images of 10 humans (five females and five males; age range, 21–46 years) from the IXI dataset (https://brain-development.org/ixi-dataset/), 10 chimpanzees (*Pan troglodytes*; five females and five males, age range; 13–43 years) from the National Chimpanzee Brain Resource (NS092988; http://www.chimpanzeebrain.org), and 10 Japanese macaques (*Macaca fuscata*, sex and age not available) from the National Institute of Physiological Sciences, Okazaki, Japan. Slice interval and pixel size were 1.2 and 0.94 mm, respectively, for humans; 0.6–0.9 and 0.6–0.9 mm, respectively, for chimpanzees; and 0.81 and 0.82 mm, respectively, for macaques. The macaque specimens were identified as adults by comparison with the magnetic resonance dataset of macaque brain development (Scott et al. 2016).

### Homologous neuroanatomical labels of the brain

To achieve neuroanatomically homologous brain transformations for the systematic investigation of brain differences among the three species, we used a well-established neuroanatomically labeled brain atlas (standard anatomical template) that had been published for each species (Rolls et al. 2020 for the human brain, Vickery et al. 2020 for the chimpanzee brain, and Rohlfing et al. 2012 for the macaque brain). The representative brains of humans, chimpanzees, and macaques were originally parcellated into 162, 130, and 724 structural regions, respectively. While these finer structural parcellations do not necessarily correspond across species, the broader divisions of the brain regions align well with sulci and gyri, displaying consistent patterns across species. To facilitate comparison and enable neuroanatomically homologous brain transformations, we consolidated the finer subdivisions into 39 neuroanatomically equivalent structural regions (Supplementary Information Fig. S1, Tables S1-3). Although both human and chimpanzee brains have distinct superior and middle temporal sulci, the macaque brain is known to possess only the longitudinal superior temporal sulcus, which corresponds to the former. However, an ambiguous, intermittent sulcus corresponding to the middle temporal sulcus has been observed in the macaque brain (von Bonin and Bailey, 1947), and the macaque brain atlas incorporates this feature in its parcellation. Similarly, while both human and chimpanzee brains have superior and inferior frontal sulci, these are absent in the macaque brain. However, the macaque brain atlas identifies the upper limb of the arcuate sulcus and its anterior extension, as well as the principal sulcus, as analogous features in its parcellation, as also described by Kobayashi et al. (2014). Furthermore, the macaque brain lacks distinct precentral and postcentral sulci, but neuroanatomically homologous regions in the frontal lobe were defined in the macaque atlas using specific reference lines. These include a line extending upward from the lower limb of the arcuate sulcus, passing through the superior prefrontal dimple, and reaching the longitudinal fissure, and another line extending downward from the upper end of the marginal sulcus on the medial surface of the cerebral hemisphere, passing through the rostral ending of the intraparietal sulcus, and reaching the lateral sulcus (von Bonin and Bailey, 1947).

Each hemisphere comprises 18 regions including 14 cerebral cortex regions, two cerebellar cortex regions, limbic system regions (hippocampus and amygdala), and the cerebral basal ganglia, in addition to the cerebellar vermis, brainstem, and white matter regions. However, some adjustments were necessary to ensure accurate neuroanatomical homology. For instance, the anterior inferior frontal gyrus in the chimpanzee atlas was incorporated into the orbitofrontal region of the frontal lobe, following Zilles and Amunts (2018). Additionally, the caudal portions of the posterior middle temporal gyrus and angular gyrus in the chimpanzee atlas, corresponding to the extrastriate cortex, were manually delineated using ITK-SNAP software (Yushkevich et al. 2006; http://www.itksnap.org) based on the intraparietal, lateral, superior temporal, and middle temporal sulci and reassigned to the superior/middle occipital lobe regions, as described Bailey et al. (1950). In the macaque atlas, the opercular regions folded within the lateral sulcus were separated from the postcentral gyrus, precentral gyrus, and supramarginal gyrus as described by Evrard (2019) and reclassified under the operculum and insula. Meanwhile, the angular gyrus was reassigned to the superior/middle occipital lobe to align with the extrastriate cortex, following von Bonin and Bailey (1947). Consequently, the lunate sulcus is not regarded as the anterior border of the occipital region in the chimpanzee and macaque brains in this study, allowing for the definition of the extrastriate cortex as the homologous region of the occipital lobe. These modifications were essential for achieving a consistent framework of neuroanatomical homology across species (Supplementary Information Fig. S1, Table S1-3).

The cerebellar vermis and brainstem were not labeled in the human and chimpanzee atlases; however, we manually segmented the corresponding regions to create the labels. The caudal border of the brainstem was defined as the margin of the foramen magnum. The white matter region was determined with reference to the segmentation of the brain image, defining anatomical labels (Ashburner and Friston 2005). The labels were visually inspected for possible inaccuracies associated with borders between regions as well as between regions and backgrounds, and manually corrected where necessary.

### Parcellation of individual brains

**Fig. 1:**
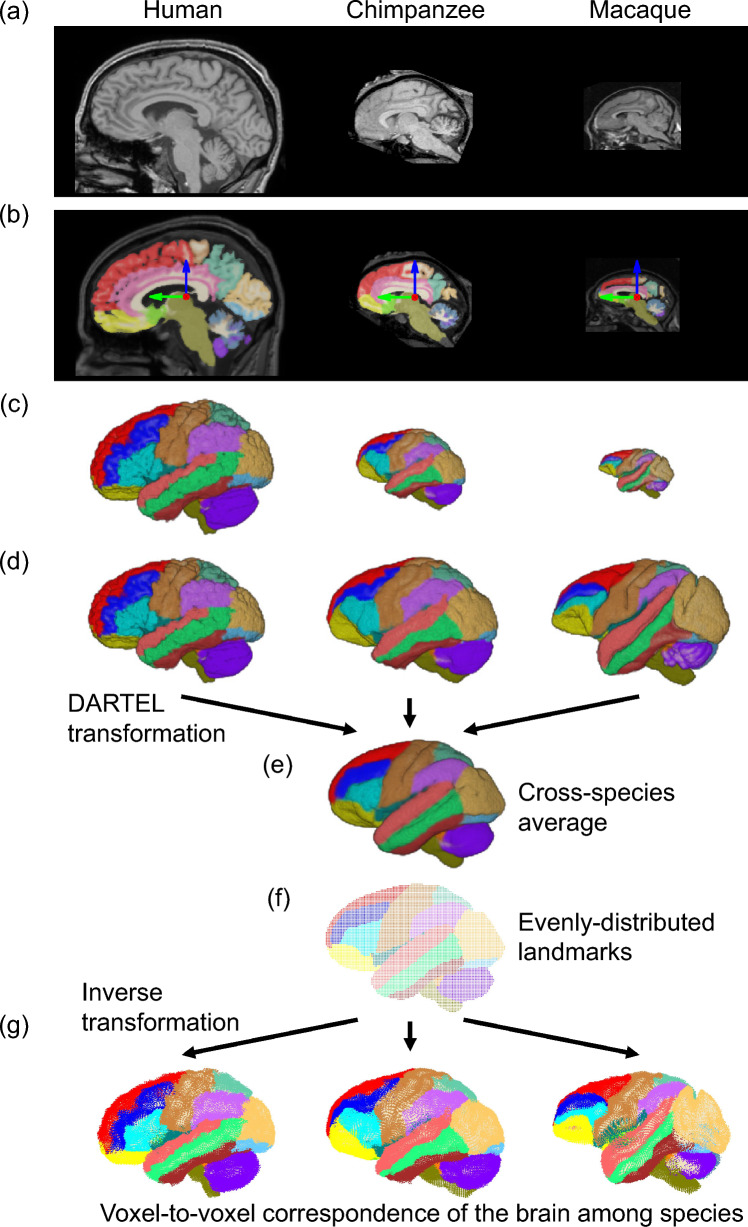
Parcellation of brains and construction of a cross-species average template brain. **a** Original cranial MRI data from humans, chimpanzees, and macaques. **b** Parcellation and spatial alignment to a reference brain space (MNI space) by a rigid-body transformation. **c** 3D representation of the parcellated, aligned brains. **d** Size normalization and rotational and skew transformation in the sagittal plane. **e** Cross-species average template brain calculated based on a spatial normalization procedure using DARTEL. **f** Evenly distributed landmarks defined on the cross-species average template brain. **g** The landmarks were mapped to each individual brain using homologous brain transformation

Individual brain images (Fig. 1a) were parcellated into 39 structural regions using the constructed neuroanatomical labels. Specifically, brain images of the individuals and those annotated with neuroanatomical labels were first aligned to a reference brain space (MNI space) using a rigid-body transformation calculated based on a unified segmentation procedure (Ashburner and Friston 2005) (Fig. 1b). The transformed brain images were resampled with isotropic voxel sizes of 1.5 × 1.5 × 1.5 mm for humans, and 0.5 × 0.5 × 0.5 mm for chimpanzees and macaques.

Using the transformed individual brain images, the species-specific population average template brain was calculated as described by Ashburner and Friston (2009). For each voxel, the average intensity value across all registered images was calculated, and an initial population-averaged template brain was generated. Individual brain images were then deformed to match the initial template, and the population-averaged template brain was updated. By repeating this process, a finely detailed, species-specific, population-averaged template brain that captures subtle anatomical variations across a population can be calculated. In addition, the nonlinear deformation functions of individual brain images that precisely match the anatomy of the template brain can be calculated based on the DARTEL algorithm—a large deformation diffeomorphic transformation that can represent smooth, topology-preserving, and invertible deformations (Ashburner 2007). If this is achieved, the inverse function can be obtained. Using these deformation functions, the homologous brain atlas was mapped to each individual brain using the template brain, and the individual brains were parcellated into 39 structural regions (Fig. 1c).

### Cross-species average template brain

To achieve neuroanatomically homologous brain transformations among humans, chimpanzees, and macaques, it is necessary to create a template brain for the three species while preserving the interspecies neuroanatomical correspondence of the parcellated regions. However, this is a complex and challenging task that requires special consideration because of the distinct anatomical differences in the brain size, shape, and structure among the three species. To account for this, the individual brain image was first adjusted by a scaling factor of the cube root of the brain volume such that the volume of all brains was standardized to match the average volume of 10 human brains (1245 cc) (Fig. 1d). After filtered by a Gaussian filter (σ = 5 pixels), the images were then registered to each other by a rigid-body transformation in the sagittal plane. To maintain the anatomical correspondence between regions, the sum of the squares of voxel intensity differences between individual and representative brain images was independently calculated for each of the 39 parcellated regions, and the sum of these differences was minimized. Subsequently, the average intensity across all registered images was calculated to obtain the initial target brain. The individual brain image was then re-registered to this target brain by using a linear affine transformation (scaling, rotation, and skew in the sagittal plane) with the same objective function so that the brains from the three species were aligned in roughly the same spatial position, orientation, and configuration (Fig. 1d).

We then further transformed and averaged the images using a nonlinear transformation based on the calculation method outlined above, which involved generating an average-shaped brain using DARTEL (Fig. 1e), as described previously (Ashburner and Friston 2009). Specifically, to avoid local minima, we performed the DARTEL transformation to the images filtered using a Gaussian filter (σ = 5 pixels) for 30 iterations, and then additional 30 iterations were performed to the unfiltered images. Thus, the cross-species average template brain was calculated considering the differences in the size and shape of each structural region. The same objective function with regularization of the smoothness of the deformation field represented by the elastic energy (Ashburner 2007) was used in this calculation.

Using deformation functions, the homologous transformation of the brain of one species to another can be calculated via the cross-species template brain, allowing the systematic investigation of similarities and differences in brain anatomy, structure, or function across different species.

### Region-based morphometry

The volumes of the subdivided brain regions in each individual brain were measured for interspecific comparisons based on the parcellated brain images. However, owing to the large size difference in the brain among the three species, each parcellated volume was normalized to the whole-brain volume (the sum of the 39 regions) for size-adjusted comparisons. The volumes of the bisymmetrical brain regions on both the left and right sides were combined, and regional volume differences were statistically evaluated using analysis of variance across 21 regions. We employed Bonferroni correction for multiple comparisons, with the adjusted *p* value set at < 0.0024 (0.05/21).

To quantify the interspecies differences and variations in regional brain volumes, we used VBM (Wright et al. 1995; Ashburner and Friston 2000; Good et al. 2001). To generate the cross-species average template brain, we obtained a deformation field (a combination of linear affine and nonlinear DARTEL transformations) that allows an individual brain image to be precisely transformed into the cross-species average template brain while preserving structural homology. Therefore, voxel-to-voxel correspondence across individual brain images was established. The spatial derivatives (Jacobian matrix) of this deformation function describe how each voxel in an individual image stretches, shears, and rotates on the cross-species average template brain, and the determinant of this Jacobian matrix represents the relative change in volume. The inverse of this value at each voxel was statistically examined for all voxels in the cross-species average brain to identify the locations in the brain with significant interspecies differences.

Here, we used a two-sample *t* test on a voxel-by-voxel basis to compare interspecific differences in anatomical brain structures. The voxel values constituted the statistical parametric map of the *t* statistics. We applied multiple comparison correction based on Random Field Theory and family wise error rate correction at the peak level. The statistical height threshold was set at *p* < 0.017 (0.05/3) over the whole brain (Friston et al. 1994, 1996; Worsley et al. 1996) because the Bonferroni correction must be employed for multiple comparisons among the three species.

### Visualization of homologous brain transformation

To visualize the neuroanatomically homologous transformation of the brain among species, a landmark-based 3D geometric morphometric technique was employed (Adams et al. 2004; Bookstein 1991; Mitteroecker and Gunz 2009; O’Higgins, 2000; Slice 2005). The center of each voxel constituting the cross-species averaged template brain was treated as a landmark, resulting in the even distribution of 511,909 landmarks across the entire brain region (Fig. 1f). The landmarks were mapped to each individual brain using the inverse of the above deformation function to obtain the coordinates of the landmarks that corresponded to those in the average brain (Fig. 1g). An average set of landmarks was calculated for each species. To visualize the transformation between the two average sets of landmarks, we initially computed the principal components (PC) of the brain landmarks using a variance–covariance matrix. Within this PC space, the three average brains were depicted as points. Subsequently, we determined the brains corresponding to the points along a linear trajectory from one brain to another. This facilitated the visualization of neuroanatomically homologous brain transformations between humans and chimpanzees; chimpanzees and macaques; and macaques and humans.

### Validation

To demonstrate the validity of the proposed neuroanatomically homologous brain transformation, the averaged cross-species template brain was inversely transformed into a representative individual brain for each species. The volume of each parcellated region was calculated and compared with the corresponding true value, which was calculated based on species-specific neuroanatomical labels. The differences were presented as a percentage of the total brain volume.

### Software

The above-described image-processing routines were performed using SPM12 revision 7771 (http://www.fil.ion.ucl.ac.uk/spm) implemented in MATLAB R2023a (MathWorks, Natick, MA, USA).

## Results

**Fig. 2:**
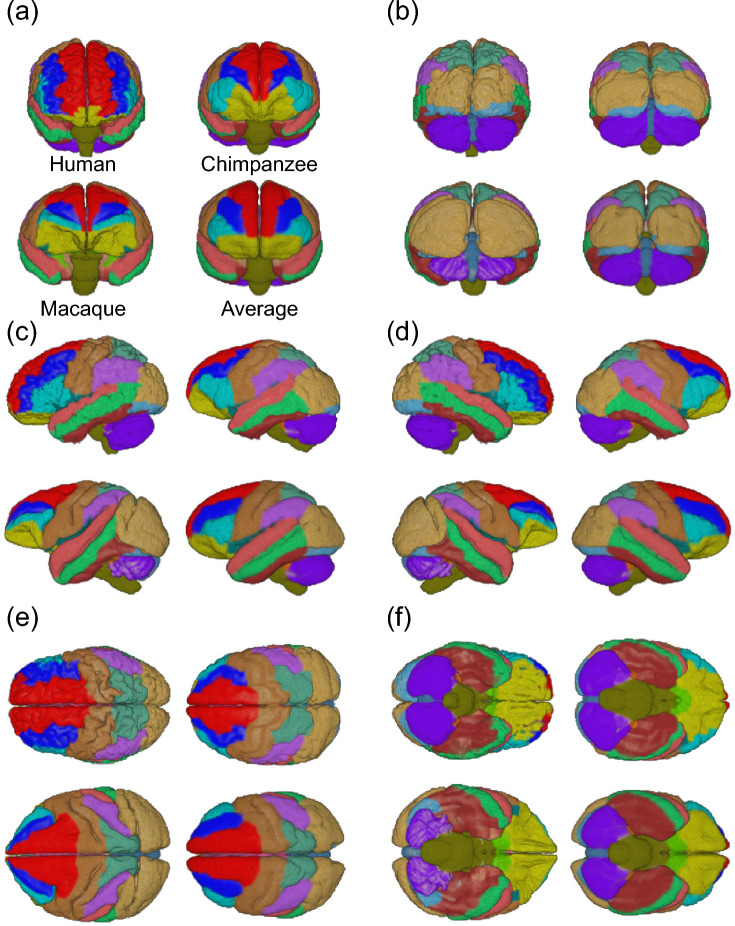
Cross-species average template brain in comparisons with the representative human, chimpanzee, and macaque brains. **a** Anterior, **b** posterior, **c** left, **d** right, **e** superior and **f** inferior views

**Fig. 3:**
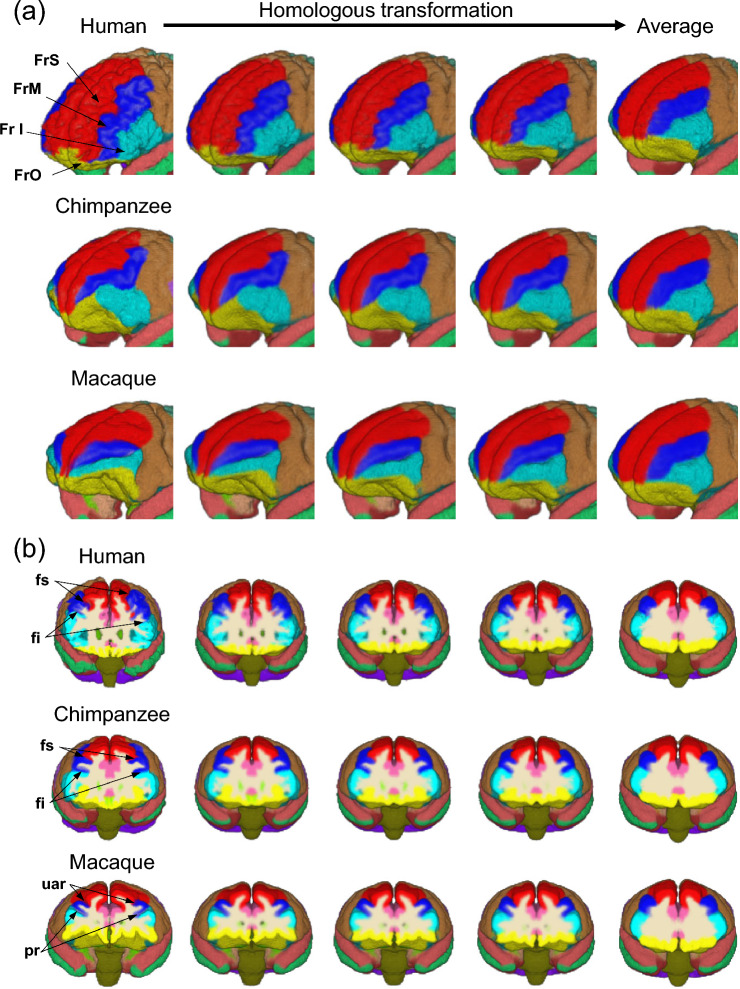
Comparisons of the topographic relationship between the frontal lobe inferior region (inferior frontal gyrus) and its adjacent brain regions before and after transformation. **a** External surface. **b** Coronal cross-section approximately at the midpoint of the frontal lobe. fs, superior frontal sulcus (corresponding to the upper limb of the arcuate sulcus, uar, in the macaque brain); fi, inferior frontal sulcus (corresponding to the principal sulcus, pr, in the macaque brain)

Figure 2 depicts the calculated cross-species average template brain, along with the parcellated brains of a representative human, chimpanzee, and macaque. Here we defined 39 neuroanatomically equivalent structural regions based on the brain atlases (See Materials and Methods). The average cross-species template brain had an intermediate shape among the three species. Figure 3 shows how the frontal lobe regions of the three species were homologously transformed into a cross-species average template brain. Using the proposed method, the transformation function from each individual brain of the three species to the cross-species average template brain was successfully achieved while preserving the morphological correspondence of each parcellated region. Table 1 shows the accuracy of the estimated volumes for each parcellated region, which are presented as a percentage of the total brain volume. The mean and standard deviations of the errors associated with estimating the regional brain volumes of the representative human, chimpanzee, and macaque were 0.06% ± 0.06%, 0.09% ± 0.11%, and 0.10% ± 0.15%, respectively, indicating that the proposed method achieved homologous cross-species brain transformation with reasonable accuracy (Table 1).

**Table 1:** Accuracy of the volume estimation for each parcellated brain region by the homologous transformation of the cross-species average template brain, represented as a percentage of total brain volume

Region	Subregion	Error (%)		
		Human	Chimpanzee	Macaque
Frontal	Superior regions	0.13	0.03	0.01
	Middle regions	0.03	0.09	0.03
	Inferior regions	0.05	0.01	0.07
	Orbitofrontal regions	0.12	0.00	0.06
Sensorimotor		0.16	0.24	0.06
Parietal	Superior/Inferior regions	0.00	0.14	0.08
	Temporoparietal regions	0.02	0.03	0.10
Temporal	Superior regions	0.06	0.08	0.17
	Middle regions	0.05	0.03	0.13
	Inferior regions	0.01	0.06	0.04
Occipital	Superior/Middle regions	0.06	0.36	0.38
	Inferior regions	0.11	0.08	0.06
Operculum and insula		0.02	0.06	0.06
Cingulate cortex		0.06	0.05	0.01
Hippocampus and amygdala		0.03	0.01	0.04
Basal ganglia		0.01	0.11	0.08
Cerebellum	Anterior parts	0.01	0.03	0.03
	Posterior parts	0.07	0.09	0.00
	Vermis	0.01	0.01	0.06
Brainstem		0.03	0.03	0.05
White matter		0.21	0.42	0.65

The means and standard deviations of the absolute volumes of the parcellated brain regions in humans, chimpanzees, and macaques are presented in Table 2. As the bilateral differences were generally very small (Table 2) and this study did not focus on the left–right differences, we included the volumes of the left and right sides for further interspecific comparisons.

**Table 2:** Mean and standard deviations of the absolute volumes of the 39 parcellated brain regions of humans, chimpanzees, and macaques

Region	Subregion	Left/Right	Volume (cc)		
			Human	Chimpanzee	Macaque
Frontal	Superior regions	Left	36.1 ± 3.1	6.1 ± 0.9	1.5 ± 0.2
		Right	34.6 ± 3.1	6.0 ± 0.7	1.5 ± 0.2
	Middle regions	Left	18.9 ± 1.5	3.9 ± 0.5	0.5 ± 0.1
		Right	19.5 ± 1.6	4.1 ± 0.5	0.5 ± 0.1
	Inferior regions	Left	15.9 ± 0.9	3.8 ± 0.5	0.4 ± 0.1
		Right	16.2 ± 0.9	3.8 ± 0.5	0.4 ± 0.1
	Orbitofrontal regions	Left	13.8 ± 1.4	4.8 ± 0.6	1.2 ± 0.1
		Right	15.1 ± 1.4	4.9 ± 0.5	1.2 ± 0.1
Sensorimotor		Left	28.2 ± 1.5	7.9 ± 1.2	1.9 ± 0.2
		Right	25.4 ± 1.4	8.4 ± 1.3	1.9 ± 0.2
Parietal	Superior/Inferior regions	Left	20.6 ± 2.1	4.6 ± 0.5	0.9 ± 0.1
		Right	20.3 ± 1.8	4.9 ± 0.6	0.9 ± 0.1
	Temporoparietal regions	Left	20.0 ± 2.1	4.0 ± 0.5	0.8 ± 0.1
		Right	19.2 ± 1.9	4.0 ± 0.5	0.8 ± 0.1
Temporal	Superior regions	Left	17.7 ± 1.5	5.4 ± 0.7	1.8 ± 0.2
		Right	19.6 ± 2.0	5.5 ± 0.7	1.7 ± 0.2
	Middle regions	Left	24.6 ± 2.2	4.3 ± 0.6	1.0 ± 0.1
		Right	24.1 ± 2.0	4.3 ± 0.6	1.0 ± 0.1
	Inferior regions	Left	28.9 ± 2.0	7.7 ± 1.1	1.3 ± 0.2
		Right	30.5 ± 2.4	7.9 ± 1.0	1.4 ± 0.2
Occipital	Superior/Middle regions	Left	31.1 ± 3.7	10.9 ± 1.1	2.7 ± 0.3
		Right	26.6 ± 3.2	10.9 ± 1.1	2.7 ± 0.3
	Inferior regions	Left	12.4 ± 1.0	3.1 ± 0.4	0.7 ± 0.1
		Right	11.7 ± 1.1	3.0 ± 0.3	0.7 ± 0.1
Operculum and insula		Left	13.9 ± 1.0	3.7 ± 0.5	0.9 ± 0.1
		Right	14.0 ± 1.1	3.6 ± 0.5	0.9 ± 0.1
Cingulate cortex		Left	14.9 ± 1.2	4.3 ± 0.5	0.9 ± 0.1
		Right	16.3 ± 1.2	4.9 ± 0.6	1.0 ± 0.1
Hippocampus and amygdala		Left	6.1 ± 0.5	2.3 ± 0.3	0.9 ± 0.1
		Right	5.9 ± 0.5	2.2 ± 0.3	0.9 ± 0.1
Basal ganglia		Left	15.3 ± 1.1	5.5 ± 0.6	2.5 ± 0.3
		Right	15.1 ± 1.2	5.6 ± 0.6	2.5 ± 0.3
Cerebellum	Anterior parts	Left	5.9 ± 0.5	2.2 ± 0.3	0.4 ± 0.0
		Right	5.0 ± 0.4	2.2 ± 0.2	0.4 ± 0.0
	Posterior parts	Left	47.1 ± 3.2	13.2 ± 2.2	1.2 ± 0.1
		Right	46.7 ± 3.4	13.1 ± 2.0	1.2 ± 0.1
	Vermis		8.0 ± 0.3	2.4 ± 0.3	1.5 ± 0.2
Brainstem			48.4 ± 3.4	19.9 ± 2.0	6.6 ± 0.6
White matter			452.0 ± 33.7	132.0 ± 12.6	29.0 ± 2.8
Total			1245.5 ± 87.3	351.2 ± 33.9	80.2 ± 8.4

Data are presented as mean ± standard deviation

**Fig. 4:**
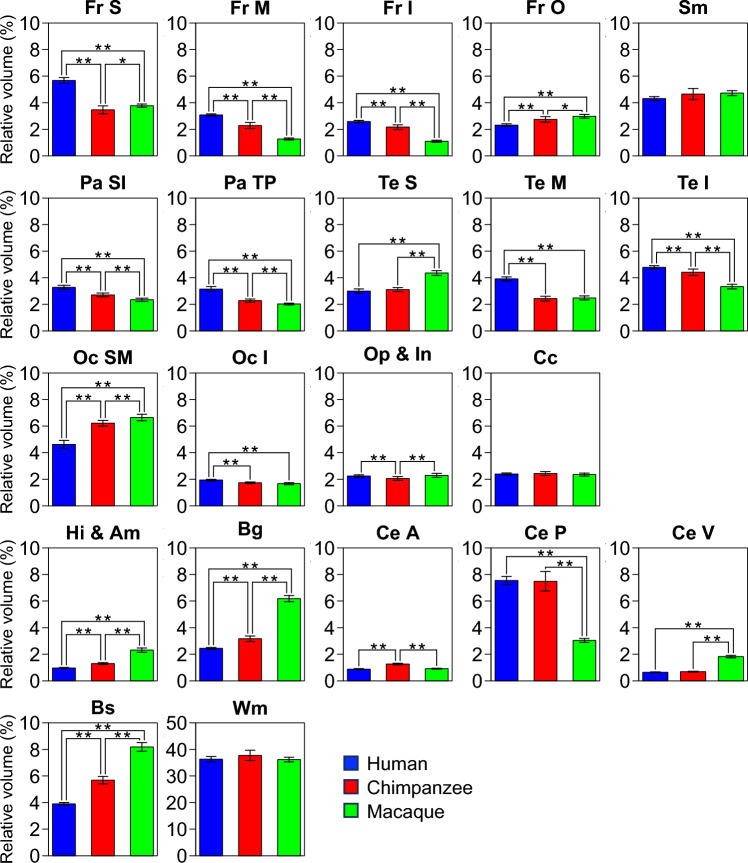
Comparisons of the relative volumes of the parcellated brain regions among humans, chimpanzees, and macaques. Each parcellated volume was normalized to the whole-brain volume for size-adjusted comparisons. The regionally relative specific volume differences were evaluated by analysis of variance across 21 parcellated regions. We employed Bonferroni correction for multiple comparisons; the threshold of *p* < 0.0024 (= 0.05/21) has been set for statistical significance. Fr, frontal lobe; Pa, parietal lobe; Te, temporal lobe; Oc, occipital lobe; Ce, cerebellum; Sm, sensorimotor cortex; Op, operculum; In, insula; Cc, cingulate cortex; Hi, hippocampus; Am, amygdala; Bg, basal ganglia; Bs, brainstem; Wm, white matter; S, superior region; M, middle region; I, inferior region; O, orbitofrontal region; SI, superior and inferior region; TP, temporoparietal junction; A, anterior region; P, posterior region; V, vermis

Figure 4 shows the relative volumes of each parcellated region for the three species. The superior, middle, and inferior regions of the frontal lobe; superior and inferior regions; temporoparietal junction of the parietal lobe; and middle and inferior regions of the temporal lobe in humans were significantly larger than those of the other two species. In macaques, however, the superior and middle regions of the occipital lobe, limbic system (hippocampus and amygdala), and basal ganglia were significantly larger than those in the other two species. The relative volumes of the chimpanzees were between the human and macaque values. Regarding the cerebellum, the vermis is significantly larger in macaques than in humans or chimpanzees. However, the posterior region of the cerebellum is significantly larger in both humans and chimpanzees. The relative volumes of the sensorimotor cortex, cingulate cortex, and white matter remained unchanged in the three species.

**Fig. 5:**
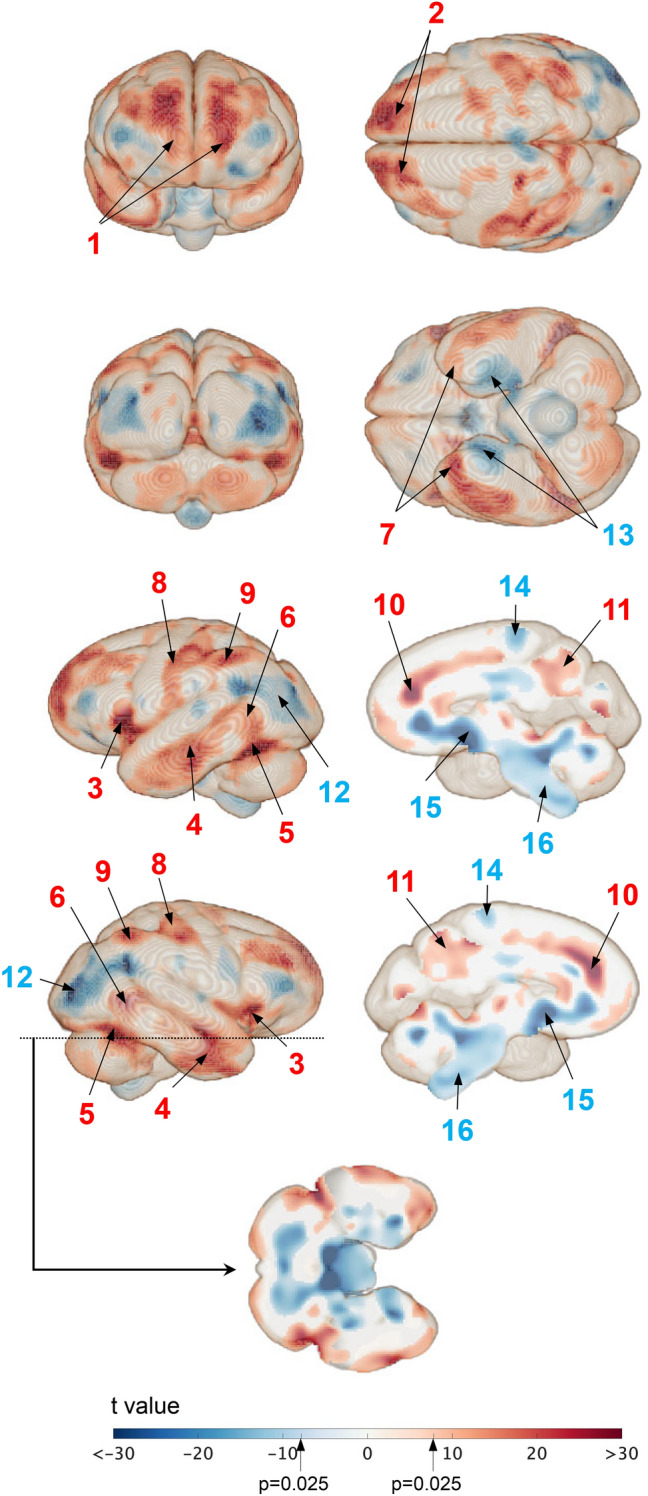
Voxel-wise volume differences of the cortical surface of the brain between humans and chimpanzees. The surface area where the differences are statistically significant (*p* < 0.05, with family-wise error correction) are in red, if expanded, and in blue, if shrunken, in humans

Figure 5 presents the results of the VBM analysis, with significant voxel-wise volume differences in the cortical surface of the brain between humans and chimpanzees (Supplementary Information, movie files). The homologous transformation of the parcellated brain visualized that there were significant relative volumetric expansions in the (1) frontal pole (BA10; for reference, the corresponding Brodmann areas [BA] are presented), (2) dorsolateral prefrontal cortex (DLPC; BA9, 46), (3) posteroinferior frontal gyrus (BA44, 45), (4) middle temporal gyrus (BA21), (5) posteroinferior temporal gyrus (fusiform gyrus, BA37), (6) temporoparietal junction (TPJ; BA39,40), (7) temporal pole (BA38), (8) primary somatosensory cortex (BA3,1,2), (9) angular gyrus (BA39), (10) anterior cingulate cortex (ACC; medial prefrontal cortex, BA24,32), and (11) precuneus (BA7) in humans compared to those in chimpanzees, while a significant relative volumetric shrinkage was observed in the (12) superior and middle occipital lobes, (13) parahippocampal gyrus (BA34–36), (14) primary motor cortex (area innervating leg muscles; BA4), (15) basal forebrain, and (16) human brainstem.

**Fig. 6:**
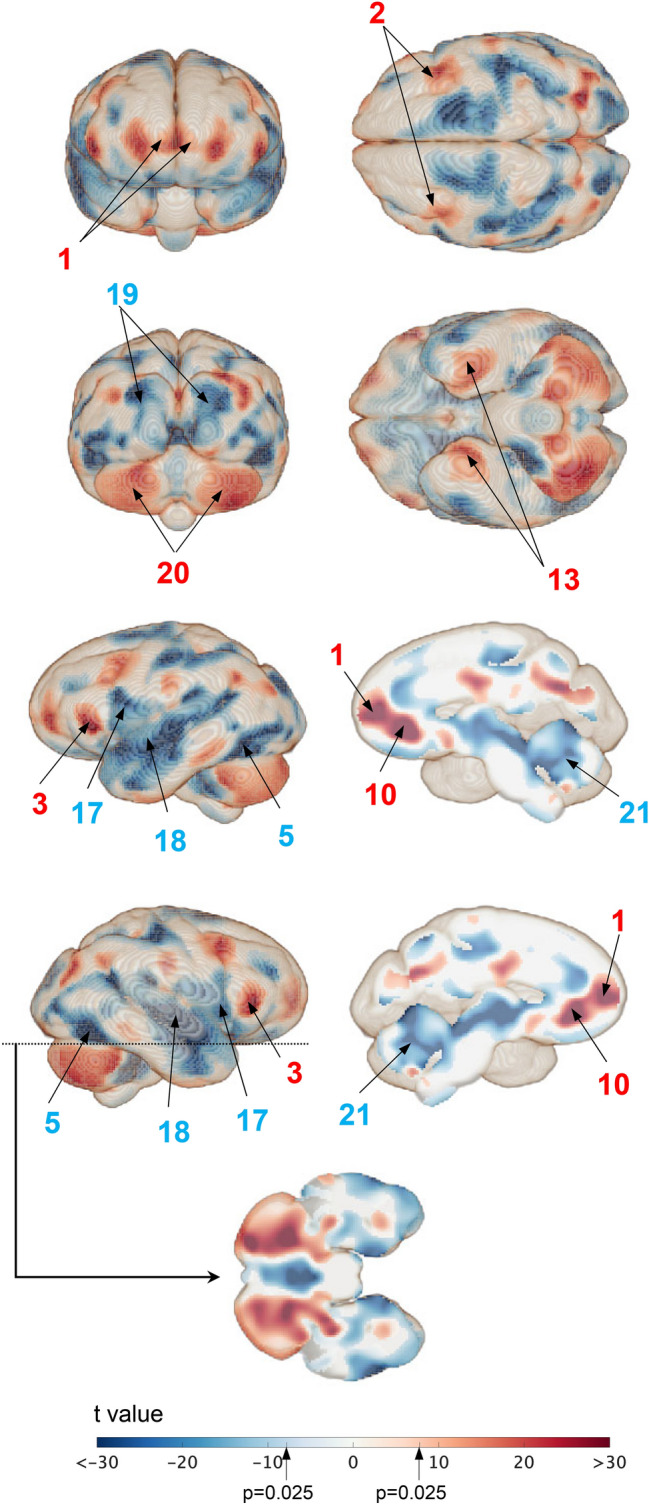
Voxel-wise volume differences of the cortical surface of the brain between chimpanzees and macaques. The surface area where the differences are statistically significant (*p* < 0.05, with family-wise error correction) are in red, if expanded, and in blue, if shrunken, in chimpanzees

Figure 6 presents the results of the VBM analysis, which shows significant voxel-wise volume differences in the cortical surface of the brain between chimpanzees and macaques (Supplementary Information, movie files). Compared to macaques, chimpanzees had a significantly larger (1) frontal pole, (2) DLPC, (3) posteroinferior frontal gyrus, (13) parahippocampal gyrus, and (10) ACC as well as a significantly smaller (17) inferolateral primary motor cortex (area innervating the head and neck muscles), (18) superior temporal gyrus, and (5) posteroinferior temporal gyrus (Fig. 6).

## Discussion

The present study showed that, compared to chimpanzees, humans had a significantly larger and more expanded prefrontal cortex (frontal lobe excluding the precentral gyrus, i.e., FrS, FrM, and FrI), particularly the frontal pole (#1), DPFC (#2), and posteroinferior frontal gyrus (#3; Figs. 4 and 5), which has been reported previously (Schoenemann et al. 2005; Smaers et al 2017; Semendeferi et al. 2001; Schenker et al. 2010). The prefrontal cortex is responsible for executive function (Miyake et al. 2000), which comprises a set of cognitive functions that allow individuals to plan, organize, and regulate behavior, and make decisions to complete tasks and achieve goals. The frontal pole is reportedly involved in multi-task coordination (Koechlin et al. 1999; Gilbert, et al. 2006) and episodic memory (Umeda et al. 2005), and is associated with working memory (Barbey, 2013; Fuster 2000) and probabilistic inference (Donoso et al. 2014) which are key components of executive function. Furthermore, the DPFC is a crucial cortical area for tool making (Putt et al. 2019; Stout et al. 2015). The posteroinferior frontal gyrus is involved in speech production and language comprehension (Horwitz et al. 2003). The enlargement of the gross proportion of a specific cortical region is assumed to correspond to an increase in the behavioral and cognitive capacity associated with the region because the size of a brain region reflects its neuron count and connectivity, serving as a proxy for its computational capacity (Striedter 2005). Consequently, individuals with larger volumes in specific areas are expected to perform related cognitive tasks more efficiently (e.g., Pohlack et al. 2014; Yuan and Raz 2014). Therefore, considerable expansion of the prefrontal cortex may allow humans to possess higher social cognitive abilities, as well as unique skills in toolmaking and language.

Furthermore, the present study showed that the temporal lobes (TeM and TeI), particularly the middle temporal gyrus (#4) and posteroinferior temporal gyrus (#5), were significantly larger and more expanded in humans than in chimpanzees (Figs. 4 and 5). The middle temporal lobe plays crucial roles in language, such as semantic comprehension (Binder et al. 2009), and the fusiform gyrus is involved in face recognition (Kanwisher et al. 1997). Compared to chimpanzees, humans had significantly larger parietal lobes, excluding the postcentral gyrus (i.e., PaSI and PaTP). Furthermore, the cortical surfaces of the angular gyrus (#9) and the precuneus (#11) expanded more in humans than in chimpanzees (Figs. 4 and 5). The angular gyrus is related to calculation ability, attention, and decision-making (Seghier 2013; Studer et al. 2014). The precuneus is the functional core of the default model network (Utevsky et al. 2014), and plays an important role in visuospatial integration and egocentric memory (Margulies et al. 2009). Therefore, the temporal and parietal lobes have expanded significantly in humans, particularly in the regions that are responsible for advanced social cognitive functions.

Interestingly, in the present study, compared with chimpanzees, humans had a significantly expanded TPJ (#6), temporal pole (#7), and ACC (#10) (Figs. 4 and 5). These areas are associated with mentalizing (Frith and Frith 2003), which refers to the cognitive ability to read the mental states of others, such as intentions, emotions, and desires (Frith and Frith 2006), and plays a fundamental role in human social cognition and communication because it allows the contextual differentiation of one’s own mind from that of others to understand the mental states of others. It serves as a neural foundation for unique prosocial human behaviors, including cooperation, empathy, and reciprocity. This ability could, therefore, have increased the chance of survival in humans who lived in socially complex societies. Furthermore, these three areas (TPJ, temporal pole, and ACC) are associated with self–other discrimination processes and theory of mind (Saxe and Kanwisher 2003), face recognition, semantic and socio-emotional processing (Herlin et al. 2021), and motivation, sociability, and error monitoring (Apps et al. 2016), respectively. Our findings suggest that these functional components are vital for high-order cognitive functions, such as mentalizing. In contrast, compared to humans, chimpanzees had a significantly larger occipital lobe (OcSM) and visual cortex (#12), as reported previously (de Sousa et al. 2010; Holloway 1992).

In the macaque brain, subcortical regions that are responsible for basic survival functions, namely the limbic system (hippocampus + amygdala), basal ganglia, and brainstem, were relatively larger than in human and chimpanzee brains (Figs. 4 and 6). This is consistent with the significant surface expansion of the parahippocampal gyrus (#13), which is involved in complex emotional processes owing to the interconnection between the amygdala and other limbic systems (Lew and Semendeferi 2017). However, our results are not consistent with previous reports of relatively larger hippocampus and amygdala in humans (Barger et al. 2014), likely due to differences in size normalization methods. In the present study, whole brain volume including the cerebellum was used for normalization, whereas Barger et al. (2014) excluded the cerebellum, which is proportionally larger in humans. What is unique to the macaque is that it has a significantly larger superior temporal lobe (Te S; #18) and superior and middle occipital lobes (Oc SM; #19) compared to the other two species, as also reported previously (Rilling and Seligman 2002; deSousa et al., 2010). The temporal and parietal lobes correspond to the auditory and visual cortices, respectively, which are responsible for basic sensory perception but probably contribute less to advanced cognitive abilities.

The posterior cerebellar hemispheres (Ce P; #20) were significantly more stretched in chimpanzees and humans than in macaques, whereas the opposite was noted for the vermis (Ce V; #21), as demonstrated by MacLeod et al. (2003) previously (Figs. 4–6). The vermis is responsible for maintaining balance and posture as well as coordinating and regulating motor functions. However, recently, the cerebellar hemispheres have been identified as being responsible for not only motor control and learning but also higher cognitive functions, such as language, working memory, and executive functions (Desmond and Fiez 1998; Ito 2008; Marvel and Desmond 2010; Kochiyama et al. 2018a, b). Cerebellar enlargement in both humans and chimpanzees could be linked to their higher social complexity, compared to that in macaques.

The present study proposes a method to homologously transform the brain among the three species by calculating an average template brain, as is frequently done in computational neuroanatomy (Kochiyama et al. 2018a, b). Such an average template brain allows the calculation of a deformation function from an individual brain to the template brain, as well as its inverse function, based on the DARTEL algorithm, because it involves a diffeomorphic function. Therefore, calculating the average template brain enabled the transformation of one brain into another through the template brain. A challenge in the present study was to create a neuroanatomically homologous cross-species average template brain that accounts for the large interspecific variability in whole-brain and local brain morphologies, that is, differences in position, size, and shape of each brain region among species. To solve these problems, we introduced an affine transformation of individual brains before performing diffeomorphic registration of the images based on the DARTEL algorithm, allowing a more precise registration of brain images from three different species. In addition, we calculated the diffeomorphic registration of the whole brain by independently evaluating the registrations of 39 segmented brain regions based on brain atlases to facilitate region-to-region homologous transformations within the brain. With these attempts, the cross-species average template brain and the neuroanatomically homologous interspecific transformation of the brain across the three species was successfully achieved, while preserving the anatomical correspondence of each brain region.

One limitation of the present study is the small number of specimens used for each species (10 per species). However, the interspecific variability in brain morphology among the three species is much greater than that within a single species. Therefore, we believe that the small sample size for each species did not affect the results of this study. Another limitation pertains to the assumptions underlying the anatomical labels and regional boundaries. The brain atlases used in this study were parcellated based on macroscopic gyral and sulcal morphology, which, while generally corresponding to cytoarchitectural areas (White et al. 1997), may not perfectly align with their underlying cytoarchitectural structure. We acknowledge that some of these regional boundaries are based on assumptions that may currently lack extensive empirical support. Nonetheless, we believe that these boundaries represent the most reasonable approximation given the available evidence. Future updates to brain atlases, potentially enabled by emerging technologies such as ultra-high-field MRI, may refine these definitions. Such advancements could provide an opportunity to revisit and further validate the conclusions of this study. Finally, in the present study, perfect registration and transformation of the brain were not guaranteed because of the spatial resolution of the MRI data and the existence of local minima when attempting to volumetrically match the complex cortical folding patterns. Although such errors were generally small, as demonstrated in the present study, it might be possible to improve the accuracy; therefore, the reliability of the present results by combining the surface-based approach (Fischl and Dale 2000) with the current voxel-based registration technique needs validation because the spherical topology of the cortical surface is more suitable when dealing with the registration of cortical folding patterns. Such efforts to improve the accuracy of the registration and volumetric representation of the brain by combining 2D and 3D approaches should be investigated to achieve more detailed interspecific comparisons of the brain with improved accuracy.

In conclusion, the present study successfully proposed a method to homologously transform the brain and applied the proposed technique to extract and visualize cortical and subcortical brain reorganization that occurred during the evolution of human, chimpanzee, and macaque brains. The human prefrontal cortex exhibits significant enlargement, which possibly suggests associations with executive function, toolmaking skills, and unique language capabilities. In addition, the temporal and parietal lobes, notably associated with mentalization, show a marked expansion in humans, which emphasizes their advanced social cognitive functions.

## Supplementary Information

Below is the link to the electronic supplementary material.

Supplementary file1 (DOCX 912 KB)

Supplementary file2 (MP4 2426 KB)

Supplementary file3 (MP4 2623 KB)

Supplementary file4 (MP4 2702 KB)

## Data Availability

The human MRI data from the IXI Dataset (http://brain-development.org/ixi-dataset/) and the chimpanzee MRI data from the National Chimpanzee Brain Resource (http://www.chimpanzeebrain.org) are available from the websites. The macaque MRI data from the National Institute of Physiological Sciences are available from the corresponding authors upon reasonable request. The other data that support the findings of this study are available from the corresponding author upon reasonable request.
