# Supplementary material for: Enlargement of the human prefrontal cortex and brain mentalizing network: anatomically homogenous cross-species brain transformation
Source: Brain Struct Funct. 2025 Jan 24;230(2):34. doi: 10.1007/s00429-025-02896-7 (PMC11762074; doi:10.1007/s00429-025-02896-7)
Supplement: Supplementary file 1 — Supplementary file1 (DOCX 912 KB) [file 429_2025_2896_MOESM1_ESM.docx]

**Supporting Information for**

***Enlargement of the human prefrontal cortex and brain mentalizing network: anatomically homogenous cross-species brain transformation***

Hideki Amano^1*^, Hiroki C. Tanabe^2^, and Naomichi Ogihara^1*^

1 Department of Biological Sciences, Graduate School of Science, The University of Tokyo, Tokyo, 113-0033, Japan

2 Department of Cognitive and Psychological Sciences, Graduate School of Informatics, Nagoya University, Nagoya, 464-8601, Japan

*Corresponding authors: Naomichi Ogihara and Hideki Amano

**Email:**  ogihara@bs.s.u-tokyo.ac.jp, hideki_amano@bs.s.u-tokyo.ac.jp

**This PDF file includes:**

Fig. S1

Tables S1 to S3

Legends for Movies S1 to S3

**Other supporting materials for this manuscript include the following:**

Movies S1 to S3

**Fig. S1** Neuroanatomical labels used in the present study. The human (a), chimpanzee (b) and macaque (c) labels were based on AAL3 (Rolls et al., 2020), Jena130 (Vickery et al., 2020), and INIA19 (Rohlfing et al., 2012), respectively. Fr, frontal lobe; Pa, parietal lobe; Te, temporal lobe; Oc, occipital lobe; Ce, cerebellum; Sm, sensorimotor cortex; Op, operculum; In, insula; Cc, cingulate cortex; Hi, hippocampus; Am, amygdala; Bg, basal ganglia; Bs, brainstem; Wm, white matter; S, superior region; M, middle region; I, inferior region; O, orbitofrontal region; SI, superior and inferior region; TP, temporoparietal junction; A, anterior region; P, posterior region; V, vermis. See Supplementary Information Tables S1–3 for correspondence between the anatomical labeling in the atlases and the 39 parcellated brain regions in the present study.

The sulci representing the divisions of the 39 regions are also shown (solid lines). The dotted lines represent the boundaries used by the atlases to define these divisions. fs, superior frontal sulcus; fi, inferior frontal sulcus; fid, inferior frontal dimple, fo; fronto-orbital sulcus; pr, principal sulcus; uar, upper limb of arcuate sulcus; lar, lower limb of arcuate sulcus; pc, precentral sulcus; pcL, line corresponding to precentral sulcus; pa, paracentral sulcus, psd, superior prefrontal dimple; pt, postcentral sulcus; ip, intraparietal sulcus; la, lateral sulcus; ts, superior temporal sulcus; tm, middle temporal sulcus; oci, inferior occipital sulcus; ci, cingulate sulcus; ma, marginal sulcus; su, subparietal sulcus; po, parieto-occipital sulcus; ca, calcarine sulcus, foL, line marking the border between the FrO and other frontal regions; uarL, extended line from the anterior end of the arcuate sulcus; mpcL, line marking the border between the FrS and Sm on the medial surface; ptL, line corresponding to the postcentral sulcus; laL, horizontal line starting from the horizontal branch of the lateral sulcus; ptoL, line marking the border between the parietal/temporal region and the occipital region.

**Table S1**. Correspondence between the anatomical labelling in the AAL3 atlas for human brains and the 39 parcellated brain regions.

| Region | Subregion | Left/  Right | Region name of AAL3 atlas | Abbre-  viation |
| --- | --- | --- | --- | --- |
| Frontal | Superior region | X | Frontal_Sup, Supp_Motor_Area, Frontal_Sup_Medial | Fr S |
|  | Middle region | X | Frontal_Mid | Fr M |
|  | Inferior region | X | Frontal_Inf_Oper, Frontal_Inf_Tri, Frontal_Inf_Orb | Fr I |
|  | Orbitofrontal region | X | Frontal_Med_Orb, OFCmed, OFCant, OFCpost, OFClat, Rectus | Fr O |
| Sensory-motor |  | X | Precentral, Postcentral, Paracentral_Lobule | Sm |
| Parietal | Superior/Inferior region | X | Parietal_Sup, Precuneus | Pa SI |
|  | Temporo-parietal region | X | SupraMarginal, Angular, Parietal_Inf | Pa TP |
| Temporal | Superior region | X | Temporal_Sup, Temporal_Pole_Sup, Heschl | Te S |
|  | Middle region | X | Temporal_Mid, Temporal_Pole_Mid | Te M |
|  | Inferior region | X | Temporal_Inf, ParaHippocampal, Fusiform | Te I |
| Occipital | Superior/Middle region | X | Calcarine, Cuneus, Occipital_Sup, Occipital_Mid | Oc SM |
|  | Inferior region | X | Occipital_inf, Lingual | Oc I |
| Operculum & Insula |  | X | Rolandic_Oper, Insula | Op&In |
| Cingulate cortex |  | X | ACC_sub, ACC_pre, ACC_sup | Cc |
| Hippocampus & Amygdala |  | X | Hippocampus, Amygdala | Hi&Am |
| Basal ganglia |  | X | Olfactory, Caudate, Putamen, Pallidum, N_Acc | Bg |
| Cerebellum | Anterior part | X | Cerebellum_3, Cerebellum_4_5 | Ce A |
|  | Posterior part | X | Cerebellum_Crus1, Cerebellum_Crus2, Cerebellum_6, Cerebellum_7b,  Cerebellum_8  Cerebellum_9, Cerebellum_10 | Ce P |
|  | Vermis |  | Vermis_1_2, Vermis_3, Vermis_4_5, Vermis_6, Vermis_7, Vermis_8, Vermis_9, Vermis_10 | Ce V |
| Brainstem |  |  | Thal_AV, Thal_LP, Thal_VA, Thal_VL, Thal_VPL, hal_IL, Thal_Re, Thal_MDm, Thal_MDl, Thal_LGN, Thal_MGN, Thal_PuI, Thal_PuM, Thal_PuA, Thal_PuL, VTA, SN_pc, SN_pr, Red_N, LC, Raphe_D, Raphe_M | Bs |
| White matter |  |  |  | Wm |

**Table S2**. Correspondence between the anatomical labelling in the Davi130 atlas for chimpanzee brains and the 39 parcellated brain regions.

| Region | Subregion | Left/  Right | Region name of Davi130 atlas | Abbre-  viation |
| --- | --- | --- | --- | --- |
| Frontal | Superior region | X | Anterior_Superior_Frontal_Gyrus,  Middle_Superior_Frontal_Gyrus, Posterior_Superior_Frontal_Gyrus | Fr S |
|  | Middle region | X | Anterior_Middle_Frontal_Gyrus, Posterior_Middle_Frontal_Gyrus | Fr M |
|  | Inferior region | X | Middle_Inferior_Frontal_Gyrus, Posterior_Inferior_Frontal_Gyrus | Fr I |
|  | Orbitofrontal region | X | Medial_Orbitofrontal_Cortex,  Lateral_Orbito-frontal_Cortex,  ^(1)^ Anterior_Inferior_Frontal_Gyrus | Fr O |
| Sensory-motor |  | X | Superior_Precentral_Gyrus, Middle_Precentral_Gyrus, Inferior_Precentral_Gyrus,  Paracentral_Lobul,  Superior_Postcentral_Gyrus  Middle_Postcentral_Gyrus, Inferior_Postcentral_Gyrus | Sm |
| Parietal | Superior/Inferior region | X | Superior_Parietal_Lobule, Precuneus | Pa SI |
|  | Temporo-parietal region | X | Supramarginal_Gyrus, ^(2)^ Angular_Gyrus | Pa TP |
| Temporal | Superior region | X | Anterior_Transverse_Temporal_Gyrus, Posterior_Transverse_Temporal_Gyrus, Anterior_Superior_Temporal_Gyrus, sterior_Superior_Temporal_Gyrus | Te S |
|  | Middle region | X | Anterior_Middle_Temporal_Gyrus,  ^(2)^ Posterior_Middle_Temporal_Gyrus | Te M |
|  | Inferior region | X | Anterior_Inferior_Temporal_Gyrus, Posterior_Inferior_Temporal_Gyru, Anterior_Fusiform_Gyrus, Posterior_Fusiform_Gyrus, Parahippocampal_Gyrus  Entorhinal_Cortex | Te I |
| Occipital | Superior/Middle region | X | Cuneus, Calcarine_Area, Superior_Occipital_Gyrus, Middle_Occipital_Gyrus | Oc SM |
|  | Inferior region | X | Lingual_Gyrus,  Inferior_Occipital_Gyrus | Oc I |
| Operculum & Insula |  | X | Frontal_Operculum,  Partietal_Operculum,  Anterior_Insula,  Posterior_Insula | Op&In |
| Cingulate cortex |  | X | Anterior_Cingulate_Cortex, Middle_Cingulate_Cortex, Posterior_Cingulate_Cortex | Cc |
| Hippocampus & Amygdala |  | X | Hippocampus, Amygdala | Hi&Am |
| Basal ganglia |  | X | Caudate_Nuclues,  Nucleus_Accumbens,  Basal_Forebrain_Nuclei,  Putamen, Globus_pallidus | Bg |
| Cerebellum | Anterior part | X | Cerebellum_II-Anterior_Quadrangulate_  Lobe,  Cerebellum_III-Anterior_Quadrangulate  _Lobe,  Cerebellum_IV-Anterior_Quadrangulate  _Lobe | Ce A |
| Cerebellum | Posterior part | X | Cerebellum_V-Anterior_Lobe_A,  Cerebellum_V-Anterior_Lobe_B,  Cerebellum_VI-Superior_Posterior_  Lobe,  Cerebellum_VIIA-Superior_Posterior_  Lobe-Crus_II_of_Ansiform_Lobule_  with_Paramedian_1,  Cerebellum_VIIA-Superior_Posterior_  Lobe-Crus_I_of_Ansiform_Lobule  Cerebellum_VIIIAB-Inferior_Posterior_  Lobe-PML, Cerebellum_IX-Tonsil | Ce P |
|  | Vermis |  |  | Ce V |
| Brainstem |  |  | Thalamus | Bs |
| White matter |  |  |  | Wm |

1. The "Anterior_Inferior_Frontal_Gyrus," originally defined as part of the inferior frontal gyrus in the original atlas, was included in the "Orbitofrontal regions" in the present study. This change was made because this region corresponds more closely to a part of the "Frontal Orbitofrontal regions" in humans and macaques (Zilles and Amunts, 2018).
2. The posterior parts of the "Angular_Gyrus" and "Posterior_Middle_Temporal_Gyrus," which correspond to the extrastriate cortex in the occipital lobe (Bailey, von Bonin, and McCulloch, 1950), were manually separated and included in the "Occipital Superior/Middle regions" in the present study. The border for this separation was defined by a line connecting the posterior ends of the intraparietal sulcus, lateral sulcus, superior temporal sulcus, and middle temporal sulcus.

**Table S3**. Correspondence between the anatomical labelling in the Inia 19 atlas for macaque brains and the 39 parcellated brain regions.

| Region | Subregion | Left/  Right | Region name of Inia19 atlas | Abbre-  viation |
| --- | --- | --- | --- | --- |
| Frontal | Superior region | X | superior_frontal_gyrus | Fr S |
|  | Middle region | X | middle_frontal_gyrus | Fr M |
|  | Inferior region | X | inferior_frontal_gyrus | Fr I |
|  | Orbitofrontal region | X | fronto-orbital_gyrus, lateral_orbital_gyrus, straight_gyrus,  medial_orbital_gyrus | Fr O |
| Sensory-motor |  | X | ^(1)^ postcentral_gyrus, ^(1)^ precentral_gyrus | Sm |
| Parietal | Superior/Inferior region | X | precuneus, superior_parietal_lobule | Pa SI |
|  | Temporo-parietal region | X | ^(1)^ supramarginal_gyrus | Pa TP |
| Temporal | Superior region | X | superior_temporal_gyrus | Te S |
|  | Middle region | X | middle_temporal_gyrus | Te M |
|  | Inferior region | X | fusiform_gyrus, inferior_temporal_gyrus, posterior_parahippocampal_gyrus, parasubicular_area, presubiculum, entorhinal_area | Te I |
| Occipital | Superior/Middle region | X | occipital_gyrus, cuneus,  annectant_gyrus, ^(2)^ angular_gyrus | Oc SM |
|  | Inferior region | X | lingual_gyrus, inferior_occipital_gyrus | Oc I |
| Operculum & Insula |  | X | insula | Op&In |
| Cingulate cortex |  | X | posterior_cingulate_gyrus, isthmus_of_the_cingulate_gyrus, anterior_cingulate_gyrus, callosal_sulcus | Cc |
| Hippocampus & Amygdala |  | X | fasciolar_gyrus, alveus,  lateral_amygdalar_nucleus, accessory_basal_nucleus_of_the_  amygdala,  basal_nucleus_of_the_amygdala  claustral_amygdalar_area, eriamygdalar_area, stratum_radiatum,  paralaminar_nucleus_of_the_amygdala, granular_layer_of_the_dentate_gyrus  molecular_layer_of_the_dentate_gyrus, subiculum,  stratum_pyramidale_of_the_CA1_field, stratum_pyramidale_of_the_CA3_field,  stratum_pyramidale_of_the_CA2_field, hilus_of_the_dentate_gyrus, amygdalohippocampal_area, CA1_field, hippocampalamygdaloid_transition_area  anterior_amygdalar_area, cortical_amygdalar_nucleus,  amygdala_-_not_otherwise_specified | Hi&Am |
| Basal ganglia |  | X | caudate_nucleus, putamen, claustrum, lateral_medullary_lamina, putamen, claustrum, lateral_medullary_lamina, lateral_globus_pallidus  medial_medullary_lamina, nucleus_of_stria_terminalis, lateral_septal_nucleus, nucleus_  accumbens, prepyriform_area  olfactory_tubercle, ansa_lenticularis,  substantia_innominata, medial_septal_nucleus, dorsal_septal_nucleus  nucleus_of_the_diagonal_band, endopiriform_nucleus, central_amygdalar_nucleus, medial_amygdalar_nucleus,  inner_portion_of_the_medial_globus_  pallidus  outer_portion_of_the_medial_globus_  pallidus, basal_forebrain_nucleus, amygdalopiriform_transition_area, thalamostriate_vein | Bg |
| Cerebellum | Anterior part | X | anterior_quadrangular_lobule, alar_central_lobule | Ce A |
|  | Posterior part | X | gracile_lobule, simple_lobule, inferior_semilunar_lobule, superior_semilunar_lobule,  cerebellar_tonsil, flocculus, biventer_lobule | Ce P |
|  | Vermis |  | lingula, nodulus, uvula, lobule_II, lobule_III, lobule_IV, lobule_V, declive, folium, tuber, lobule_VIIIA, lobule_VIIIB | Ce V |
| ^(3)^ Brainstem |  |  | 11,12,13,14,15,16,17,18,19,20,22,23,24,  25, 26,29,30,31,32,33,34,35,36,38,39,  40,42,43,44,46,47,49,50,53, ,56,59,60,  62,64,66,67,68,72,76,77,78,82,83,84,88,  92,93,95,96,102,103,104,105,106,107,  108,109,113,114,115,116,117,118,119,  120,121,122,123,124,126,128,129,130,  131,132,133,134,136,137,138,139,140,  141,144,145,154,156,157,158,159,163,  164,165,169,170,175,176,177,178,179,  180,181,183,186,195,198,200,255,256,  257,258,259,260,263,265,266,268,269,  270,273,274,275,277,278,279,281,284,  285,286,287,292,298,300,301,302,308,  310,311,315,317,318,321,323,324,325,  326,327,328,330,331,332,333,335,336,  366,368,369,370,371,372,373,374,379,  381,383,384,386,388,391,394,395,396,  397,398,399,402,403,404,405,406,413,  414,415,416,418,419,420,421,425,426,  427,429,430,432,433,435,437,438,441,  445,497,499,500,503,509,513,536,583,  585,589,605 and corresponding numbers in the right side. | Bs |
| White matter |  |  |  | Wm |

1. The regions folded within the lateral sulcus, corresponding to the operculum, were manually separated and included in the "Operculum & Insula" regions.
2. The "angular_gyrus," originally defined as part of the parietal lobe in the original atlas, was reclassified under the "Occipital Superior/Middle regions" in this study. This change was made because this region aligns more closely with the extrastriate cortex in the occipital lobe (von Bonin and Bailey, 1948).
3. Region numbers as defined in the INIA19 atlas are listed in place of region names.

**References**

Bailey, P., von Bonin, & G., McCulloch, W.S. (1950). The Isocortex of the Chimpanzee. The University of Illinois Press.

Rohlfing, T., Kroenke, C. D., Sullivan, E. V., Dubach, M. F., Bowden, D. M., Grant, K. A., & Pfefferbaum, A. (2012). The INIA19 template and NeuroMaps atlas for primate brain image parcellation and spatial normalization. Frontiers in Neuroinformatics, 6, 27.

Rolls, E. T., Huang, C. C., Lin, C. P., Feng, J., & Joliot, M. (2020). Automated anatomical labelling atlas 3. NeuroImage, 206, 116189.

Vickery, S., Hopkins, W. D., Sherwood, C. C., Schapiro, S. J., Latzman, R. D., Caspers, S., Gaser, C., Eickhoff, S. B., Dahnke, R., & Hoffstaedter, F. (2020). Chimpanzee brain morphometry utilizing standardized MRI preprocessing and macroanatomical annotations. eLife, 9, e60136.

von Bonin, G., & Bailey, P. (1947). The Neocortex of Macaca mulatta. The University of Illinois Press.

Zilles, K., & Amunts, K. (2018). Cytoarchitectonic and receptorarchitectonic organization in Broca's region and surrounding cortex. Current Opinion in Behavioral Sciences, 21, 93–105.

Movie S1 (separate file). Homologous transformation of the parcellated brain between human and chimpanzee

Movie S2 (separate file). Homologous transformation of the parcellated brain between chimpanzee and macaque

Movie S3 (separate file). Homologous transformation of the parcellated brain between macaque and human.
